# Late Occurrence of Lens Particle Glaucoma Due to an Occult Glass Intralenticular Foreign Body

**DOI:** 10.4103/0974-9233.53870

**Published:** 2009

**Authors:** Nadia A. Hassan, Margaret A. Reddy, Suresh S. Reddy

**Affiliations:** From the Department of Ophthalmology, Mohammed A. Rahman Al-Bahar Eye Centre, Ibn Sina Hospital, Ministry of Health, Kuwait

**Keywords:** Intralenticular Foreign Body, Lens Particle Glaucoma, Mature Cataract

## Abstract

We report a case of traumatic mature cataract with a late occurrence of lens particle glaucoma after 11 years of trauma due to a presence of an occult intralenticular glass foreign body which was detected accidentally during the cataract surgery.

## INTRODUCTION

In cases of traumatic cataract caused by projectile objects, most patients present with a definite history of ocular trauma. However, asymptomatic penetrating ocular injuries do occur; these may be ignored or under-appreciated and eventually forgotten.^1^ Lens injury is a major complication of trauma and is seen in 30% to 65% of cases.^2^ Disruption of lens capsule by penetrating foreign body or surgery, liberates lens material, which can obstruct trabecular meshwork. In rare cases, the lens material can be released long after surgery or trauma.^3^ A case of mature cataract with an occult intralenticular foreign body causing delayed onset of lens particle glaucoma is reported.

## CASE REPORT

A 22-year-old man presented to our hospital with complaints of acute pain and redness in the right eye with headache centered over the right side of the head of 2 days' duration. He gave history of trauma inflicted 11 years ago with corneal repair done at that time. No documented evidence of the trauma or surgery performed was available.

**Figure 1:**
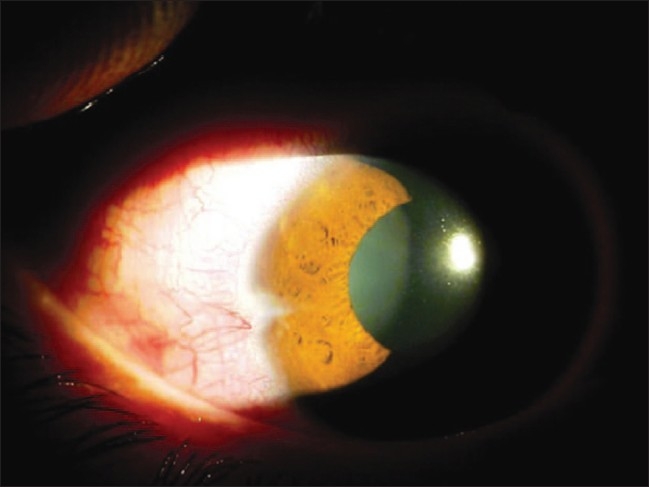
Slit-lamp photograph showing corneal scar with circulating lens particles in the anterior chamber after the eye was quietened

On examination the best corrected visual acuity was accurate perception and projection of light in the right eye and 20/20 in the left eye. The right eye showed conjunctival injection. The cornea was hazy with moderate micro-cystic edema and stromal edema. A corneal scar was seen at 9-o'clock position (Figure 1). The anterior chamber was deep with flare+2, cells+2 and circulating fluffy white lens debris. The white flocculent flaky material was deposited on the iris and the anterior capsule. The lens was densely opaque with wrinkling of the anterior capsule.

**Figure 2:**
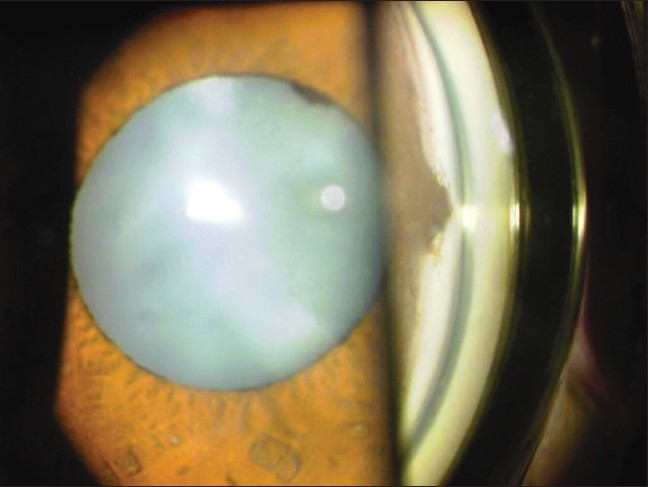
Slit-lamp photograph with gonioscopy showing mature cataract, grade IV open angle, anterior synechiae of approximately 1 clock hour at 9-o'clock position

**Figure 3:**
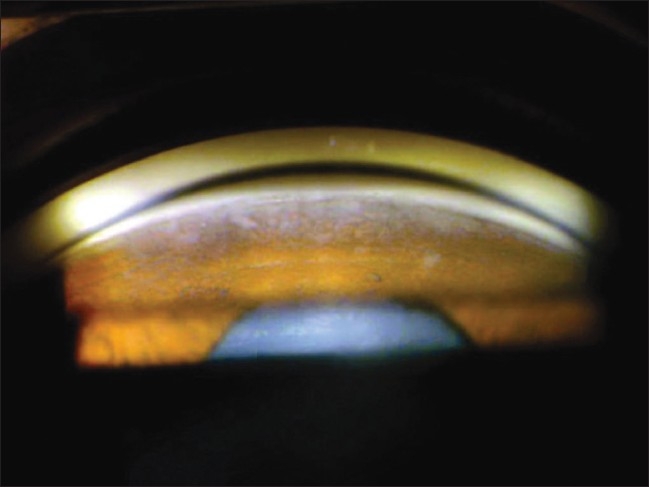
Gonioscopy showing open angle grade IV with deposition of white flaky lens material

The pupil was semi-dilated, fixed with peripheral anterior synechiae of 1 clock hour at 9-o'clock position (Figure 2). There was no evidence of tears in the iris. No keratic precipitates were seen. The intraocular pressure by applanation tonometry was 66 mm in the right eye and 14 mm in the left eye. The fundus could not be viewed due to lens opacification. Left eye fundus was normal with a healthy disc of cup-disc ratio 0.2. Results of B-scan ultrasonography were normal. Gonioscopy examination showed open angle grade IV with 360° deposition of white flaky material in the anterior chamber angle (Figure 3). There were no signs of angle recession. Left eye also revealed normal grade IV open angle.

**Figure 4:**
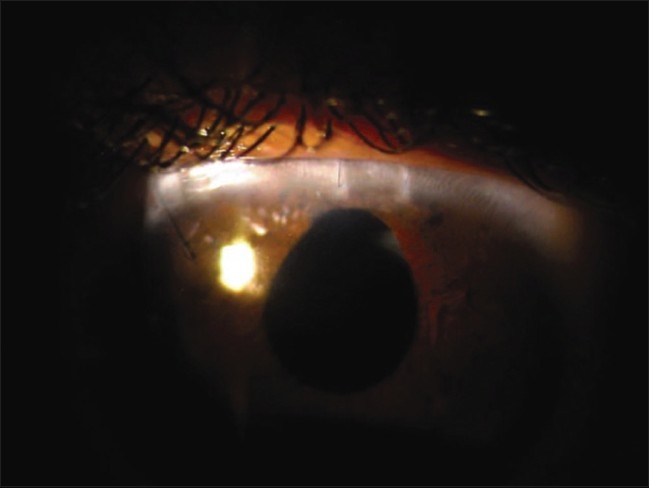
Slit-lamp photograph showing postoperative status after extraction of cataract and intralenticular foreign body

Following pupillary dilatation, slit-lamp examination of the anterior segment showed wrinkling of the anterior capsule. The status of the posterior capsule appeared intact. There was no iridodonesis or phacodonesis. In view of these findings, a diagnosis of right eye lens particle glaucoma was made.

After the IOP was moderately reduced by medical therapy with anti-glaucoma medications and 1% prednisolone acetate eye drops four times daily to control the intraocular inflammation, the patient was posted for cataract extraction with an IOL implantation after 5 days of initial presentation to our hospital. The patient was posted for phacoemulsification with implantation of IOL after the eye was quietened and pressure moderately reduced. During the surgical procedure, presence of intralenticular glass foreign body, which was 3-4 mm in size, was accidentally detected, along with the presence of posterior capsular fibrosis. The foreign body was just adjacent to the posterior capsule and slipped into the posterior segment with an extension of posterior capsular tear and vitreous prolapse while attempting removal. At this stage, a vitreo-retinal surgeon peformed a pars plana vitrectomy with removal of the foreign body and perfomed endolaser around an iatrogenic retinal tear, with injection of C3F8 gas. Due to inadequate posterior capsular support and zonular dehiscence, a scleral-fixated IOL was implanted (Figure 4). Postoperatively, the patient was prescribed 1% prednisolone acetate eye drops in tapering doses and timolol 0.5% drops twice daily. The intraocular pressure (IOP) was 20 mm in the right eye and 14 mm in the left eye. On fundus examination, the right eye showed intact retina with a cup-disc ratio of 0.5. At 3 month follow-up, the best corrected visual acuity in the operated eye was 20/20 with an IOP of 16 mm on timolol 0.5% twice daily. Aqueous humor was collected for cytologic examination before the surgery. Cytologic examination of the aqueous revealed a proteinaceous background without macrophages.

## DISCUSSION

Intralenticular foreign bodies account for approximately 10% of all intraocular foreign bodies. Clinicians should consider the possibility of an asymptomatic intraocular foreign body even if the patients have no definite history of ocular trauma. Intralenticular foreign bodies, even metallic ones, may remain inert for a long period of time.^4^

When the lens is injured, capsular integrity may be violated and may result in a visually significant cataract. In most cases, the lens becomes sufficiently opaque to require cataract extraction for visual rehabilitation.^5^ In addition, the escape of lenticular proteins and particles may result in glaucoma and severe intraocular inflammation. In this patient, there might have been a capsular injury with retention of the inert intralenticular foreign body that was likely sealed within the lens cortex. The findings in our case suggested that the patients had injury to both the anterior and posterior capsule. This patient had wrinkling of anterior capsule and a mature cataract, which suggested that anterior capsular trauma had occurred along with gradual development of a traumatic cataract and subsequent protein leakage resulting in lens particle glaucoma. Previous studies have revealed that anterior capsular trauma should be suspected when signs such as pitting of the anterior lenticular surface, surface irregularity or localized lenticular opacity, are present^6^. Coexistent localized posterior capsule tears were evident after lens aspirations in cases with intralenticular foreign bodies as noted in our case.^7^ Previous reports have suggested that posterior capsule breaks develop thick fibrous opaque margins approximately 6 weeks after trauma^6^ This report highlights the feature that even when the anterior capsule is intact, the possibility of an intraocular foreign body should not be excluded because the capsule may restore its continuity by subcapsular epithelial proliferation.^8^

The accurate detection and localization of intraocular foreign bodies is a critical component in the preoperative ophthalmologic assessment and surgical planning. To avoid such surprises as reported in this case, apart from performing an ultrasound, a CT scan is recommended. A helical CT scan is a more sensitive imaging technique for the detection of glass intraocular foreign bodies when compared with axial CT, MR imaging and sonography.^9^ A CT scan is the standard diagnostic test because it detects most intraocular foreign bodies and is safe in the presence of metallic foreign bodies.^10^ It is imperative to maintain a high index of suspicion in such cases to avoid misdiagnosis. In lens particle glaucoma, the lens protein can cause obstruction to aqueous outflow like that experienced in phacolytic glaucoma, although free particulate lens material is the major component.^11^ The cellular reaction to these lens particles may also contribute to glaucoma; however, these lens particles themselves can mechanically impair trabecular drainage.^12^ The cytologic report in this patient showed proteineaceous material without macrophages. Lens particle glaucoma resembles phacolytic glaucoma except that there is a history of trauma or surgery that releases lens proteins into the anterior chamber resulting in the obstruction of trabecular meshwork, thereby causing increase in the intraocular pressure. Generally the glaucoma has its onset a few days after the precipitating event. In rare cases, the lens material can be released long after surgery or trauma as noted in our patients.^13^
